# Supplementary material for: The Impact of New Treatments on Short- and MID-Term Outcomes in Bilateral Lung Transplant: A Propensity Score Study
Source: J Clin Med. 2022 Oct 3;11(19):5859. doi: 10.3390/jcm11195859 (PMC9571142; doi:10.3390/jcm11195859)
Supplement: Supplementary file 1 [file jcm-11-05859-s001.zip › jcm-1842367-supplementary.pdf]

SUPPLEMENTAL MATERIAL

TITLE: THE IMPACT OF NEW TREATMENTS ON SHORT- AND MID-TERM OUTCOMES IN BILATERAL LUNG TRANSPLANT: A PROPENSITY SCORE STUDY

Annalisa Boscolo MD, PhD, Andrea Dell' Amore MD, Prof, Tommaso Pettenuzzo MD, Nicolò Sella MD[c.a.], Alessandro De Cassai MD, Elisa Pistollato MD, Nicola Cacco MD, Andrea Manzan MD, Agnese De Carolis, Federico Geraldini MD, Giulia Lorenzoni PhD, Federica Pezzuto MD, Giovanni Zambello MD, Marco Schiavon MD, Prof, Fiorella Calabrese MD, Prof, Dario Gregori PhD, Prof, Emanuele Cozzi PhD, Prof, Federico Rea MD, Prof, Paolo Navalesi Prof, FERS.

Table S1. STROBE Statement-Checklist

Methods S1. Indications for VA ECMO

Table S1. STROBE Statement-Checklist

	Item No	Recommendation	Page No
**Title and abstract**	1	(*a*) Indicate the study's design with a commonly used term in the title or the abstract	1
		(*b*) Provide in the abstract an informative and balanced summary of what was done and what was found	4, 5
**Introduction**			
Background/rationale	2	Explain the scientific background and rationale for the investigation being reported	5

Objectives	3	State specific objectives, including any prespecified hypotheses	6
**Methods**			
Study design	4	Present key elements of study design early in the paper	7
Setting	5	Describe the setting, locations, and relevant dates, including periods of recruitment, exposure, follow-up, and data collection	7
Participants	6	(*a*) Give the eligibility criteria and the sources and methods of selection of participants. Describe methods of follow-up	7-8
		(*b*) For matched studies, give matching criteria and number of exposed and unexposed	--

Variables	7	Clearly define all outcomes, exposures, predictors, potential confounders, and effect modifiers. Give diagnostic criteria, if applicable	8-9
Data sources/ measurement	8	For each variable of interest, give sources of data and details of methods of assessment (measurement). Describe comparability of assessment methods if there is more than one group	8-9
Bias	9	Describe any efforts to address potential sources of bias	-
Study size	10	Explain how the study size was arrived at	Figure 1
Quantitative variables	11	Explain how quantitative variables were handled in the analyses. If applicable, describe which groupings were chosen and why	10
Statistical methods	12	(*a*) Describe all statistical methods, including those used to control for confounding	10

		(*b*) Describe any methods used to examine subgroups and interactions	10
		(*c*) Explain how missing data were addressed	10
		(*d*) If applicable, explain how loss to follow-up was addressed	-
		(*e*) Describe any sensitivity analyses	-
**Results**			
Participants	13	(a) Report numbers of individuals at each stage of study—e.g., numbers potentially eligible, examined for eligibility, confirmed eligible, included in the study, completing follow-up, and analysed	11, Figure 1
		(b) Give reasons for non-participation at each stage	Figure 1

		(c) Consider use of a flow diagram	Figure 1
Descriptive data	14	(a) Give characteristics of study participants (e.g., demographic, clinical, social) and information on exposures and potential confounders	11
		(b) Indicate number of participants with missing data for each variable of interest	-
		© Summarise follow-up time (e.g., average and total amount)	-
Outcome data	15	Report numbers of outcome events or summary measures over time	12-13
Main results	16	(a) Give unadjusted estimates and, if applicable, confounder-adjusted estimates and their precision (e.g., 95% confidence interval). Make clear which confounders were adjusted for and why they were included	12, 13<br>Figure 2,3

		(b) Report category boundaries when continuous variables were categorized<br><br>(c) If relevant, consider translating estimates of relative risk into absolute risk for a meaningful time period	
Other analyses	17	Report other analyses done—e.g., analyses of subgroups and interactions, and sensitivity analyses	-
**Discussion**			
Key results	18	Summarise key results with reference to study objectives	13-18
Limitations	19	Discuss limitations of the study, taking into account sources of potential bias or imprecision.	17, 18

		Discuss both direction and magnitude of any potential bias	
Interpretation	20	Give a cautious overall interpretation of results considering objectives, limitations, multiplicity of analyses, results from similar studies, and other relevant evidence	18
Generalisability	21	Discuss the generalisability (external validity) of the study results	-
**Other information**			
Funding	22	Give the source of funding and the role of the funders for the present study and, if applicable, for the original study on which the present article is based	-

Methods S1. Indications for VA ECMO

The main indications for:

1. ***Intraoperative 'rescue' VA-ECMO*** were pulmonary artery hypertension, low cardiac index, and severe cardiomegaly with impaired right heart function or patients in which the clamping of the first pulmonary artery or the single lung ventilation cause cardiorespiratory instability[19,20];
2. ***'Prolonged' ECMO*** in the intensive care unit (ICU) were marginal donors, long ischemic time (>7 hours), high-risk recipient (severe pulmonary artery hypertension with right ventricular impairment), severe pulmonary artery hypertension after reperfusion (>2/3 the systemic pressure), high requirement of inotropes, signs of early reperfusion lung injury with rapid worsening of the respiratory and hemodynamic parameters, and necessity of aggressive (and not 'protective') ventilation to maintain acceptable pO2, pCO2, and pH value[19,20].
